# The Concurrent Validity and Test–Retest Reliability of a Smartphone-Based Markerless System

**DOI:** 10.3390/s26123934

**Published:** 2026-06-21

**Authors:** Kristen F. Nicholson, Jared J. Duane, William Carter, Garrett Fernandez, Jakob Wolf, Robert J. Butler, Garrett S. Bullock

**Affiliations:** 1 Department of Orthopaedic Surgery, Wake Forest University School of Medicine, Winston-Salem, NC 27101, USA; 2 Department of Biomedical Engineering, Wake Forest University School of Medicine, Winston-Salem, NC 27101, USA; 3 Nashville Soccer Club, Nashville, TN 37203, USA; 4 Department of Biostatistics and Data Science, Wake Forest University School of Medicine, Winston-Salem, NC 27101, USA; 5 Centre for Sport Injury Prevention Research, University of Calgary, Calgary, AB T2N 1N4, Canada

**Keywords:** criterion validity, countermovement jump, range of motion, biomechanics

## Abstract

Increasing the accessibility and portability of precise biomechanical data to sports scientists can assist in making data-driven decisions. The purpose of this study was: (1) Assess the concurrent and convergence validity of discrete and continuous waveform biomechanics of a smartphone-based markerless system. (2) Assess test–retest reliability of the smartphone-based markerless system. Movements were recorded simultaneously with two iPhones using Uplift Labs computer vision software and with Qualisys, a 12-camera marker-based motion capture system. Each participant performed two evaluations, one week apart, consisting of two countermovement jumps. Nested Bland–Altman limits of agreement (LOA), mixed-effect linear regressions, and intraclass correlation coefficients (ICC) were calculated. Twenty participants were included [Age: 24.7 (6.6) years. Height: 178.3 (4.3) cm. Mass: 86.5 (12.4) kg. Dominant Arm (Right): 17 (85%)]. Concurrent validity (LOA: 11.8 (4.4, 19.1)) demonstrated different results compared to convergence validity (Beta: 0.87 (0.68, 1.0)) and test–retest reliability (ICC: Uplift: 95.5 (90.9, 97.8). Qualisys: 94.4 (88.5, 97.3)). Uplift demonstrated greater than the a priori-determined limits of agreement across the CMJ. However, convergence validity was acceptable. Reliability suggests Uplift could be useful for tracking performance within individual athlete sessions.

## 1. Introduction

The countermovement vertical jump (CMJ) is a commonly used assessment tool in multiple areas of human movement science, including sport and rehabilitation [1,2]. Examining the biomechanical components of vertical jumps can offer valuable insights into how an athlete’s lower half can contribute to performance through explosive strength [3], and identify inefficiencies that may help in identifying injury risk [3,4]. This type of data is highly beneficial for strength coaches and player development staff to make data-driven decisions with their athletes, specifically assessing performance enhancement and prescribing injury prevention interventions [5]. In a cross sectional study of twenty-one elite male sprinters, the maximum velocity recorded during the 60 m sprint and at various intervals (0 to 10 m, 10 to 20 m, and 20 to 30 m), was positively associated with a higher vertical CMJ [6]. In relation to injury risk, a cross-sectional study of fifty-eight young male soccer players found that the CMJ can provide insight into bilateral asymmetries by examining impulse, eccentric mean force and concentric mean force during the jump [7]. Additionally a case–control study on 136 professional soccer players showed that bilateral differences in isokinetic hamstring strength could predict injury status [8].

The present-day biomechanical CMJ evaluation relies upon a laboratory setting with a marker-based motion tracking system. These systems provide an individualized kinematic and kinetic data assessment that can be used for athlete performance enhancement and injury prevention [9,10]. Marker-based motion capture uses reflective markers with sub-millimeter accuracy to determine the position and orientation of body segments, enabling the calculation of joint positions and angles [11,12]. However, marker-based systems have several barriers to an on-field assessment: the requirement of a laboratory setting with trained operators, high cost, and a time-consuming collection and data processing [13,14]. These barriers have created a need for a mobile and user-friendly assessment device for on-field or weight room assessments.

One method that has potential for on-field use is markerless motion capture technology. Markerless motion capture utilizes sophisticated human body models, computer vision and machine learning algorithms [15]. The four key components of a markerless motion capture system include: (1) the camera systems employed, (2) body model (joint location and joint angles), (3) the image features and (4) the algorithms that determine the body’s parameters (such as shape, pose, and location) [16]. These systems typically combine arrays of 2D video cameras or depth sensors with machine learning algorithms to estimate human pose during physical activities [17]. Due to the markerless motion capture systems not requiring skin-based markers, individuals can wear their own clothes, resulting in more viable ecological data [16]. This allows for biomechanical data to be collected in everyday environments which cannot be replicated in the laboratory [18].

In recent years, there have been several developments in motion capture technology which have altered biomechanics research and its accessibility. Among these advancements are smartphone-based markerless motion capture systems. One of these systems allows researchers to collect biomechanical data during functional tasks such as a CMJ with a minimum of two cell phone devices mounted on tripods. This technology produces reports containing valuable insights to assess players or patients, aiming to evaluate performance and potentially reduce injuries. The minimum requirement of two cell phone cameras enables data collection to occur anywhere, without the need for a laboratory setting. Previous validation and reliability studies on smartphone-based markerless systems have produced varying results regarding their accuracy in tracking kinematics, particularly when monitoring specific body parts across different planes but did show notable consistency [19,20,21]. These systems showed difficulty tracking ankle flexion with the highest error occurring with the subtalar joint in the ankle [19,20,21]. Additionally, one system displayed satisfactory agreement with knee flexion during the CMJ [22].

The need for on-field athlete biomechanical testing necessitates valid and reliable portable markerless motion capture tools. Thus, there is a need to assess the validity and reliability of phone-based biomechanical applications within athletic tests and measures. Increasing the accessibility and portability of precise biomechanical data can assist in making data-driven decisions regarding performance enhancement and injury prevention. Therefore, the purpose of this study was to assess the concurrent and convergence validity and test–retest reliability of the countermovement jump between a novel two cell phone markerless motion capture technology and marker-based motion capture. Specifically, the aims were: (1) Assess the concurrent and convergence validity of discrete and continuous waveform biomechanics of a smartphone-based markerless system. (2) Assess test–retest reliability of the smartphone-based markerless system.

## 2. Materials and Methods

### 2.1. Study Design

A repeated measure test–retest study was performed. The Guidelines for Reporting Reliability and Agreement Studies (GRRAS) informed reporting of this study [23]. This study and the associated statistical analysis plan was a priori registered on Open Science Framework (https://osf.io/gj2ac, original date of access: 9 May 2024). Participants were informed of the risks and benefits of the study prior to participation. This study received approval from the University Institutional Review Board.

### 2.2. Participants

Inclusion criteria consisted of participants who: (1) have the ability to fully participate in all physical activity and exercise-related activities; (2) ages 18 to 40 years old; (3) participate in exercise at least three times a week. Exclusion criteria consisted of participants who: (1) were currently rehabilitating a musculoskeletal injury with a medical provider; (2) had medically advised activity restrictions due to injury; (3) had inner-ear-diagnosed injuries; (4) had a concussion within the last 6 months.

### 2.3. Procedures

One tester performed all measurements across all participants utilizing the standard operating procedures as described below. Prior to each testing session, the tester performed calibration of both biomechanical instruments (see below).

Demographic Data Collection: Each participant’s age, height, weight and arm dominance were recorded at the first collection.

Marker-based System: At baseline in the laboratory, three-dimensional motion data were collected using a 12-camera motion analysis system (Qualisys v2024.2, Gothenburg, Sweden). Motion data were collected at 100 Hz. Data were then processed and variables were calculated with Visual3D (HAS-Motion, Kingston, ON, Canada). These data were recognized as the gold standard in comparison to the output data from Uplift (Uplift Labs, Palo Alto, CA, USA).

Uplift Setup: Capture requires 2 cell phone devices of the same model, 2 tripods and a reliable internet connection. First, each device was paired through Bluetooth. Next, the assessment “Movement Monitor Wake” was selected. For the full “Movement Monitor Wake” protocol and script see Supplementary Materials. Then, the athlete was created in the Uplift system by inputting their birth date, height, and weight. This was followed by a complete checkerboard calibration, which began by placing the checkerboard in the area where the athlete would perform the tasks. Calibration required 6 photos to be taken of the 14′ × 19′ checkerboard at various distances and angles on each device. After the pictures were taken, the cell phones were mounted on the tripods that are 10 feet from the checkerboard. The primary camera was placed directly in front of where the exercises were taking place. The secondary camera was located to the right facing perpendicularly to the primary camera, also 10 feet away. The tester confirmed that the athlete outline was in frame. After that, the tilt of the cameras were adjusted so that the camera angle was perpendicular to the ground. Finally, one last calibration photo was taken and the 30 s time capture option was selected.

Uplift analyzed these movements and generated multiple types of reports. The system analyzed full 3D visualization of the movement through a cloud visualizer paired with high-speed video and kinematic data. Uplift Capture also measured raw data for each capture including all joint key points, generated metrics and detected events in PDF movement reports.

Warm Up and Participant Preparation: Each participant was instructed to perform a warmup of ten minutes, consisting of dynamic and static stretching exercises [24]. For full description of warm up, please see Supplementary Materials.

Marker setup: After the participant completed the warmup, 32 reflective markers were applied in the following anatomical locations: left (L) toe, right (R) toe, L medial malleoli, R medial malleoli, L lateral malleoli, R lateral malleoli, L heel, R heel, L shank, R shank, L thigh, R thigh, sacrum, L ASIS, R ASIS, R acromion, L acromion, R clavicle, L lateral epicondyle, R lateral epicondyle, L medial epicondyle, R medial epicondyle, R forearm on ulnar side, L forearm on ulnar side, L ulnar styloid, R ulnar styloid, L radial styloid, R radial styloid, L trigonum spinae of the scapula, R trigonum spinae of the scapula, L inferior angle of the scapula, R inferior angle of the scapula.

Counter Movement Jump: Prior to testing, the countermovement jump was described and then performed by the tester. The participant was allowed three practice trials prior to measurement [25]. Participants performed two reps of the countermovement vertical jump within the capture area. The countermovement jump was performed with no arm swing. Participants were instructed to perform all jumps with maximal effort. The specific jump protocols are detailed below.

- CMJ (No Arm Swing)
  ▪ Tester instructed the player to stand facing in-between the two cell phones at a 45-degree angle. Participants were instructed to have their feet at shoulder width and hands on their hips.
  ▪ Tester then selected “begin recording”.
  ▪ Tester then instructed the player to “jump as high as possible while keeping your hands on your hips.” The participant should:
    ▪ Jump as high as possible.
    ▪ Resume starting position and stand still for 3 s.
    ▪ Perform his next jump after being given clearance by the tester.
    ▪ Hands must remain on hips the entire time the player is performing the CMJ.

### 2.4. Evidence of Concurrent Validity Based on Agreement and Convergence with a Reference Measure

Evidence of concurrent validity by agreement (i.e., the degree of concordance) was examined between countermovement jump kinematics measured using a smartphone-based markerless system and the same measures assessed with three-dimensional marker-based motion capture as the reference standard [26,27]. Evidence of concurrent validity by convergence was examined through correlating the countermovement jump kinematics (target constructs) measured using a smartphone-based markerless system and the same measures assessed with three-dimensional marker-based motion capture as the reference standard [28]. Convergent validity is demonstrated when different instruments, methods, or indicators designed to measure the same underlying construct yield highly correlated results, indicating that they are capturing the same concept. Convergence evidence of validity was considered satisfactory with the lower limit of the confidence interval of the association >0.50.

### 2.5. Test–Retest Repeatability

Test–retest repeatability was assessed through reliability of measurement through correlations over two testing sessions. Testing sessions were one week apart and performed at the same time of day as the initial session.

### 2.6. Sample Size Calculation

Concurrent Validity by Agreement: Using a previously established formula for measures of agreement between clinical instrumentation [29], an acceptable mean difference of 2 degrees for CMJ kinematics, with a standard deviation of 4.3 degrees [1], and 95% confidence interval level of agreement, were used to calculate the number of participants required to assess an acceptable mean difference. A continuity correction was incorporated to account for repeated measures at the individual participant level. A total of 12 participants were required, with three trials per movement, to have an acceptable mean difference confidence interval width of 2 degrees.

Concurrent Validity by Convergence: Using a previously established formula for correlation [30], an a priori 95% confidence interval width of 0.10-determined acceptance, with an expected regression coefficient of 0.85.

Within Session Repeatability: Using a previously established formula for reliability [31], an a priori 95% confidence interval width of 0.15 was determined to be acceptable, with an expected correlation of 0.90, at a 50% probability. A total of 19 participants were required to be tested for both days.

### 2.7. Statistical Analyses

Missing data was assessed prior to data analyses. No data were missing and thus a complete case analysis was performed. For descriptive data, continuous variables were reported as mean (standard deviation) and categorical data as count (percent).

Concurrent Validity by Agreement: Bland–Altman analyses used a nested cluster data structure at the individual participant level and plots were created to assess agreement between the smartphone-based markerless motion capture system and a 12-camera marker-based motion capture system. Heteroscedasticity (i.e., where there is a relation between the mean values versus the difference) and mean bias were also examined.

Concurrent Validity by Convergence: Linear mixed-effects regressions with 95% confidence intervals (95% CI) were performed to assess convergence between the smartphone-based markerless motion capture system and a 12-camera marker-based motion capture system. All 95% CI’s were calculated with 2000 bootstrap iterations.

Test–retest Reliability: Intraclass-correlation coefficients (ICC; 2, k) with 95% confidence intervals (95% CI) were performed to assess intersession reliability of the smartphone-based markerless motion capture system. The ICC scores were graded on the following scale: <0.5 = Poor, 0.5–0.75 = Moderate, 0.75–0.9 = Good and >0.9 = Excellent [32]. Measurement error was calculated with the standard error of measure (SEM). The minimal detectable change (MDC) over two sessions was also calculated [28,33].

All analyses were performed in R 4.3.2. R Core Team (2021). R: A language and environment for statistical computing. R Foundation for Statistical Computing, Vienna, Austria. URL https://www.R-project.org/. Packages included *dplyr*, *readxl*, *SimplyAgree*, *lme4*, *ggplot2*, and *psych*.

## 3. Results

A total of 20 participants were included [Age: 24.7 (6.6) years. Height: 178.3 (4.3) cm. Mass: 86.5 (12.4) kg. Dominant Arm (Right): 17 (85%)]. Collection totaled seventy-two CMJ trials (Table 1).

### Countermovement Jump Joint Angle Concurrent Validity and Test–Retest Reliability

**Figure 1:**
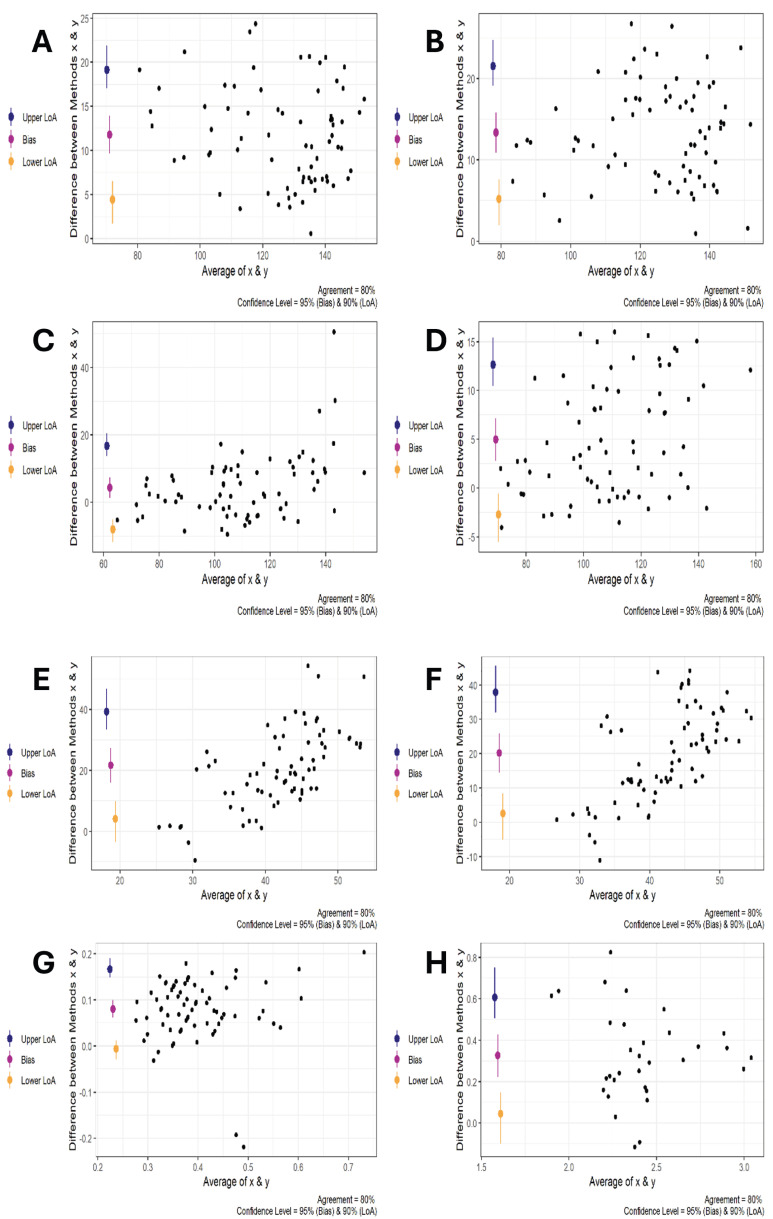
Limits of agreement for countermovement jump. Bland–Altman plot using a nested data structure (to account for data dependence generated by the repeated measures for each participant), shows differences between the mean difference in values between Uplift and three-dimensional marker-based measures, and the 95th limits of agreement (1.96 x standard error of the difference). (**A**): Right hip flexion; (**B**): Left hip flexion; (**C**): Right knee flexion; (**D**): Left knee flexion; (**E**): Right ankle dorsiflexion; (**F**): Left ankle dorsiflexion; (**G**): Jump height; (**H**): Jump velocity.

**Figure 2:**
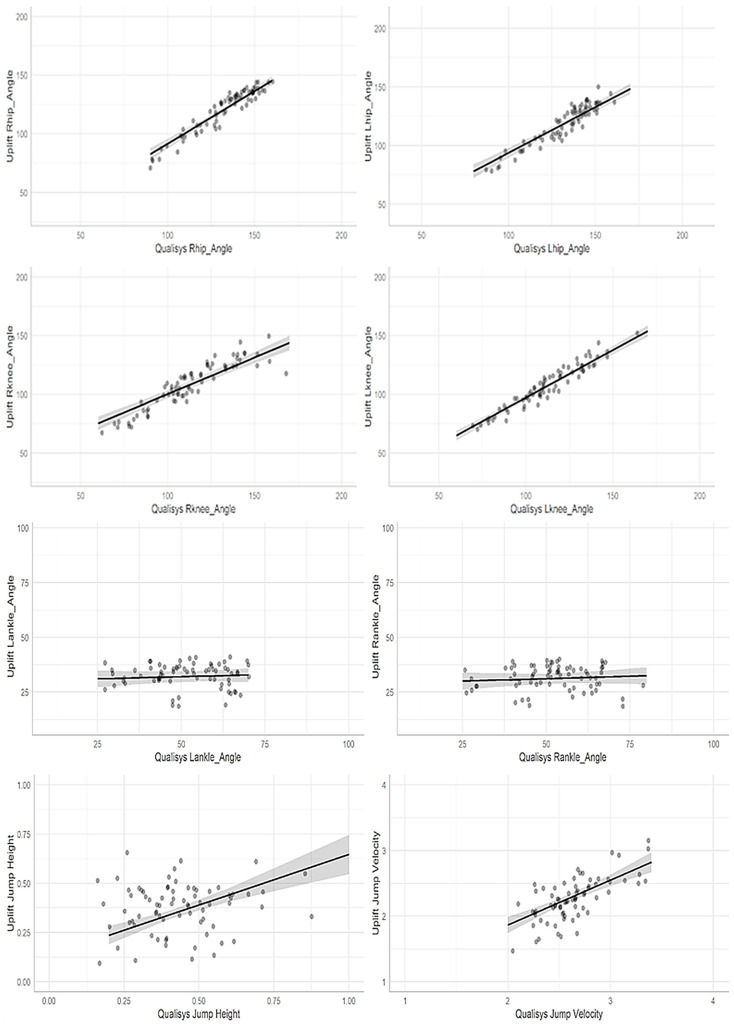
Bivariable regression plot shows convergence between Uplift and three-dimensional marker-based measures for the countermovement jump using mixed effects model linear association via regression to account for the data dependence generated by the repeated measures for each participant.

Table 2 provides the summary of variance, concurrent and convergence validity, test–retest reliability, SEM, and MDC for the CMJ. The limits of agreement across all joints ranged from two to five times above our a priori criteria of ±2° (Figure 1). The convergence (linear association) across all 95% CI lower bounds were acceptable (>0.50) except for ankle dorsiflexion (Figure 2). Test–retest reliability was similar for Uplift and marker-based 3D motion capture across all measures.

## 4. Discussion

This study examined the concurrent and convergence validity and test–retest reliability of the countermovement jump between the Uplift two smartphone-based markerless biomechanical system and the Qualisys marker-based 3D motion analysis system. Uplift’s performance in tracking maximum CMJ flexion angles was inconsistent across different segment angles. The CMJ LOA’s were not within the a priori-defined limits. However, the CMJ demonstrated similar test–retest reliability between biomechanical systems, but most joint angles did not meet the acceptable test–retest thresholds.

Limits of agreement for CMJ were beyond the a priori acceptable thresholds of ±2°. This supports previous work observing that portable markerless devices have fallen short of acceptable concurrent validity when compared to marker-based optical systems [19,34]. The wide limits of agreement across joints are interpreted as the range of body movement excursion measured by Uplift compared to 3D marker-based motion capture. To provide a practical example, if Qualisys measures knee flexion at 110° during an athlete’s CMJ, the 7° limits of agreement difference would suggest that the Uplift measurement could range from 103° to 117°. Per our a priori work with sport performance knowledge users, the maximum allowable range would be 108° to 112° in this hypothetical example. These wide limits of agreement would preclude the use of this device to create injury prevention or performance monitoring programs based on the Uplift data. However, convergence validity did meet the acceptable lower 95%-bound threshold between systems, except for ankle dorsiflexion and jump height. When observing the bivariable convergence scatter plots, the change in one unit of Uplift ranged between 0.70 and 0.90 of 3D marker-based system, except for ankle dorsiflexion and jump height point estimates. The poor agreement in ankle flexion was consistent with previous research on lower extremity joint angles tracked using smartphone-based markerless systems [34,35]. The low convergence validity and high limits of agreement for the ankle could be due to its small size, different occlusion patterns from different shoes or poor training of the reference system on this anatomical region. A possible explanation for jump height low convergence is the difficulty markerless systems face when identifying takeoff and landing phases [19]. Jump height calculation in markerless systems rely heavily on the accuracy of these key time points and even a few frames of error may impact the results. Errors in joint detection, segment mass assumptions or positioning of the individual could explain these systematic differences.

Ankle measurements demonstrated unacceptable convergence and systematic differences. Additionally, the ankle moves through a much smaller functional range during a CMJ, so a modest angular difference represents a proportionally larger measurement problem than a similar error at the hip or knee. These results do not support utilizing ankle outputs from Uplift for clinical interpretation. More broadly, they suggest that smaller joints and ranges of motion remain important limitations for smartphone-based markerless systems.

There was similar test–retest reliability, SEM, and MDC between systems across all joints. Within person variance was comparable for all systems. The test–retest results are similar to previous literature comparing two camera markerless motion capture systems [34,35]. In a validity and reliability study of 19 recreational individuals, hopping and jumping demonstrated moderate to excellent reliability (ICC = 0.70–0.94) [34]. These results suggest that the biomechanical outputs of Uplift are stable within the confines of the individual athlete.

From a clinical and performance standpoint, Uplift should be viewed as a complementary monitoring tool, not a replacement for laboratory motion capture. Its potential value lies in providing accessible and repeatable objective information when a laboratory system is unavailable and when analyzing whether the same athlete’s CMJ has changed over time. Reliable within-athlete assessment may be useful in rehabilitation, return to sport progression, or performance monitoring involving CMJs in the weight room or on field.

### Limitations

As with all studies, there are limitations. Participants with darker skin tones tend to blend in with the background at our laboratory, which could affect landmark tracking. Each participant was a different shape and size, which may change the identification of anatomical landmarks. The algorithms used to calculate the biomechanical results are proprietary and thus not available to the public, nor to the scientists who performed this study. As a result, this decreases the ability to identify the exact causes of differences between the reference standard and the markerless motion capture data. During the CMJ, hip markers could sometimes be covered. This was corrected by applying the best fit polynomial line where markers were obstructed. Variability in results between sessions is a limitation for both systems. Replicating identical capture conditions can be extremely challenging; slight changes in lighting, clothing, environment, subject positioning, or system calibration can impact outcomes. Changes in physical state may result in changes in effort level during the CMJ. Several other factors, such as sleep, soreness, and attitude, can also influence the results of the second session.

## 5. Conclusions

The concurrent validity of Uplift is specific to the movement and the joint angle examined. Uplift demonstrated greater than the a priori-determined limits of agreement across the entire CMJ, suggesting that this device should not be used to compare results between athletes concerning injury prevention or performance monitoring strategies. However, convergence validity was acceptable, except for CMJ ankle dorsiflexion. Currently, we do not recommend using Uplift as a replacement for a marker-based system, although the within-subject reliability suggests it could be useful for monitoring an individual’s countermovement jump over time.

## Figures and Tables

**Table 1:** Countermovement Jump Descriptive Statistics for Smartphone Application and Three-Dimensional Marker-based Biomechanical System.

Counter Movement Jump Discrete Kinematics	Smartphone Application	3D Marker-Based System
Right Hip Flexion CMJ	120.5° (18.6°)	132.2° (17.7°)
Left Hip Flexion CMJ	118.6° (16.8°)	131.9° (17.1°)
Right Knee Flexion CMJ	107.9° (19.3°)	112.3° (23.2°)
Left Knee Flexion CMJ	107.0° (18.4°)	111.9° (20.3°)
Right Ankle Flexion CMJ	31.8° (5.4°)	52.1° (11.8°)
Left Ankle Flexion CMJ	32.7° (5.4°)	51.7° (11.7°)
Jump Height	0.37 m (0.09)	0.44 m (0.09)
Peak Center of Mass Velocity	2.3 m/s (0.28)	2.6 m/s (0.28)

3D = 3 Dimensional; Mean (Standard Deviation); CMJ = Countermovement Jump; m = Meters; m/s = Meters per second.

**Table 2:** Countermovement Jump Joint Discrete Angle Concurrent Validity and Test–retest Reliability.

	Right Hip Flexion	Left Hip Flexion	Right Knee Flexion	Left Knee Flexion	Right Ankle Dorsiflexion	Left Ankle Dorsiflexion	Jump Height	Peak COM Jumping Velocity
Within, Between, and Combined Variance								
Within SD (95% CI)	3.1 (2.5, 3.7)	3.1 (2.4, 3.7)	6.7 (3.4, 10.0)	3.4 (2.5, 4.2)	4.4 (2.9, 6.0)	4.4 (3.2, 5.6)	0.1 (0.0, 0.1)	0.1 (0.0, 0.1)
Between SD (95% CI)	4.7 (3.9, 5.5)	5.4 (4.6, 6.2)	6.6 (4.1, 10.6)	4.8 (4.0, 5.7)	12.7 (10.5, 14.2)	12.8 (11.3, 14.2)	0.1 (0.0, 0.1)	0.2 (0.1, 0.2)
Combined SD (95% CI)	5.6 (5.0, 6.2)	6.2 (5.6, 6.9)	9.6 (7.0, 12.8)	5.9 (5.3, 6.5)	13.5 (11.4, 14.9)	13.6 (12.2, 14.8)	0.1 (0.0, 0.1)	0.2 (0.1, 0.2)
Concurrent Validity by Agreement								
Systematic Difference LOA (95% CI)	11.8 (9.7, 13.9)	13.3 (10.9, 15.8)	4.3 (1.3, 7.3)	5.0 (2.8, 7.2)	21.6 (15.9, 27.3)	20.2 (14.5, 25.9)	0.08 (0.06, 0.1)	0.33 (0.27, 0.37)
Concurrent Validity by Convergence								
Convergence	0.87 (0.68, 1.0)	0.77 (0.64, 0.88)	0.60 (0.34, 0.81)	0.80 (0.73, 0.87)	0.06 (−0.10, 0.21)	0.04 (−0.11, 0.19)	0.48 (0.16, 0.67)	0.60 (0.25, 0.85)
SD Within Uplift	18.6	16.8	19.3	18.4	5.4	5.4	0.09	0.28
SD Within Qualisys	17.7	17.1	23.2	20.3	11.8	11.7	0.09	0.28
Test–Retest Reliability								
Uplift ICC (95% CI)	95.5 (90.9, 97.8)	93.1 (85.8, 96.6)	88.6 (76.7, 94.4)	88.3 (76.1, 94.3)	83.8 (66.8, 92.1)	85.3 (69.7, 92.8)	74.5 (48.7, 87.3)	82.7 (64.8, 91.5)
Qualisys ICC (95% CI)	94.4 (88.5, 97.3)	89.8 (79.2, 95.0)	79.5 (57.8, 90.0)	87.6 (74.6, 94.0)	85.1 (51.2, 94.1)	85.5 (67.0, 93.3)	91.6 (82.6, 95.9)	92.9 (76.4, 97.2)
Uplift SEM	1.1	1.6	3.2	2.0	5.4	5.3	0.05	0.08
Qualisys SEM	1.3	2.0	4.3	2.0	5.2	5.1	0.03	0.05
Uplift MDC	3.1	4.5	8.8	5.7	15.0	14.6	0.15	0.23
Qualisys MDC	3.5	5.4	11.9	5.7	14.5	14.1	0.08	0.15

COM = Center of Mass; SD = Standard Deviation; 95% CI = 95% confidence interval; LOA = Limits of Agreement; ICC = Intraclass Correlation Coefficient; SEM = Standard Error of Measure; MDC = Minimal Detectable Change; 95% confidence intervals were calculated with 2000 bootstrap iteration.

## Data Availability

Code are openly available to ensure transparency and support future adaptation and can be accessed within the provided supplementary materials. Due to the ethical nature of the data, data can be shared by reasonable requests to the authors.

## Supplementary Materials

The following supporting information can be downloaded at https://www.mdpi.com/article/10.3390/s26123934/s1. The supplementary materials details sensitivity analyses results performed to understand the stability of the results.
