# Supplementary material for: The Concurrent Validity and Test–Retest Reliability of a Smartphone-Based Markerless System
Source: Sensors (Basel). 2026 Jun 21;26(12):3934. doi: 10.3390/s26123934 (PMC13306311; doi:10.3390/s26123934)
Supplement: Supplementary file 1 [file sensors-26-03934-s001.zip › sensors-4344459-supplementary.pdf]

```
#Reliability & Validity

#Construct Validity Analyses

library(dplyr)

library(readxl)

library(SimplyAgree)

library(lme4)

library(janitor)

library(ggplot2)

library(ggeffects)

library(irr)


######################################

#Sample size calculation


power_res <- blandPowerCurve(

  samplesizes = seq(10, 100, 1),

  mu = 0.25,

  SD = 2,

  delta = c(6,7),

  con□level = c(.90,.95),

  agree.level = c(.8,.9)

)


#Getting Sample Size

find_n(power_res, power = .8)
```

```
#Data Frame

ROM_validity <- read_excel("C:/Users/jduane/Documents/NEW WAVE/RSFMAE_NW.xlsx")




###################################################################################
#############

#Function for Limits of Agreement


check_agreement <- function(ROM_validity, y){


 if(y=="Angle"){

  #Raw Nm

  loa <- agreement_limit(x = "Difference_Qualisys",

                y = "Difference_Uplift",

                id = "Athlete",

                data = ROM_validity,

                data_type = "nest",

                agree.level = .80)

  return(list(assumptions = check(loa), #Checking Assumptions

          plot =  plot(loa, delta = 20)))  #Maximum LOA of 20 nm.

 }else if(y=="norm"){

  #Raw Nm

  loa <- agreement_limit(x = "Qualisys_norm",
```

```
              y = "Uplift_norm",
              id = "Athlete",
              data = ROM_validity,
              data_type = "nest",
              agree.level = .80)
  return(list(assumptions = check(loa), #Checking Assumptions
        plot =  plot(loa, delta = .5)))  #Maximum LOA of 0.5 BW*H.
 }


}


###########################################################################################
#Standard Error of Measure and Minimum Detectable Change Functions


# Function to calculate Standard Error of Measurement
calculate_sem <- function(sd, reliability) {
 sem <- sd * sqrt(1 - reliability)
 return(sem)
}

# Function to calculate Minimum Detectable Change
calculate_mdc <- function(sem, confidence_level = 0.95) {
```

```
  z_score <- qnorm(1 - (1 - confidence_level) / 2)
  mdc <- sem * z_score * sqrt(2)
  return(mdc)
}



###########################################################################################
#Function To Bootstrap Within and Total SD for Mixed Effects Models, For Validity of Convergence

bootMixedfunction<-function(datainput){

  library(nlme)
  # Variance of Within and Between and Overall Model
  corr_model <-lme(bootdiff~ 1, random=~1|Athlete,
            correlation=corCompSymm(form=~1|Athlete), data=datainput, na.action=na.omit)

  bootwithinsd<-as.numeric(VarCorr(corr_model)[2,2])

  bootbetweensd<-as.numeric(VarCorr(corr_model)[1,2])

  boottotalsd<-sqrt(as.numeric(VarCorr(corr_model)[1,1])+as.numeric(VarCorr(corr_model)[2,1]))

  #Convergence Linear Mixed Model
```

```
  mixed_model <- lme(Difference_Uplift ~ Difference_Qualisys, random=~1|Athlete,
                 data=datainput, na.action=na.omit)

  bootassociation <- as.numeric(summary(mixed_model)$tTable[2,1])

  combined <-cbind(bootwithinsd, bootbetweensd, boottotalsd, bootassociation)
  combined

}


bootstrap_validity_function_nm <- function(dataframe, N) {

  results <- matrix(nrow = 1,ncol = 12) #1 Row, columns for bootstrap convergence

  convergence_metrics <- data.frame() #Data frame for each bootstrap performance metrics

  #Bootstrap for loop
  for(boot in 1:N){

   #Bootstrap Sample
   boot_sample <- dataframe[sample(nrow(dataframe), replace = T), ]

   #Getting Difference Between Construct (KinaTrax) and Nextiles Elbow Varus Torque

   boot_sample$bootdiff <- boot_sample$Difference_Uplift - boot_sample$Difference_Qualisys
```

```
  #Variance of Different Paramaters Between Difference of COnstruct and Testing Technolgoy for Elbow Varus Torque

  bootwithinsd <- bootMixedfunction(boot_sample)[1] #Within Pitcher SD

  bootbetweensd <-bootMixedfunction(boot_sample)[2] #Between Pitcher SD

  boottotalsd <- bootMixedfunction(boot_sample)[3] #Combined Within & Between Pitcher SD

  bootassociation <- bootMixedfunction(boot_sample)[4]

  #Association


  convergence =  cbind(bootwithinsd, bootbetweensd, boottotalsd, bootassociation)

  convergence = data.frame(convergence)

  convergence_metrics = rbind(convergence_metrics, convergence)

  print(paste("Progress:", boot, sep=""))

 }
```

```
#Getting Aggregated Convergence Variance

#Within SD Mean

results[1, 1] <- format(round(mean(convergence_metrics[, 1], na.rm = T), 1), nsmall = 1)


#Within SD Low 95%

results[1, 2] <- format(round(quantile(convergence_metrics[, 1], probs = 0.025, na.rm = T) ,1), nsmall = 1)


#Within SD High 95%

results[1, 3] <- format(round(quantile(convergence_metrics[, 1], probs = 0.975, na.rm = T), 1), nsmall = 1)


#Between SD Mean

results[1, 4] <- format(round(mean(convergence_metrics[, 2], na.rm = T), 1), nsmall = 1)


#Between SD Low 95%

results[1, 5] <- format(round(quantile(convergence_metrics[, 2], probs = 0.025, na.rm = T), 1), nsmall = 1)


#Between SD High 95%

results[1, 6] <- format(round(quantile(convergence_metrics[, 2], probs = 0.975, na.rm = T), 1), nsmall = 1)


#Total SD Mean

results[1, 7] <- format(round(mean(convergence_metrics[, 3], na.rm = T), 1), nsmall = 1)
```

```
#Total SD Low 95%
results[1, 8] <- format(round(quantile(convergence_metrics[, 3], probs = 0.025, na.rm = T), 1), nsmall =
1)

#Total SD High 95%
results[1, 9] <- format(round(quantile(convergence_metrics[, 3], probs = 0.975, na.rm = T), 1), nsmall =1)

#Convergence Linear Beta
results[1, 10] <- format(round(mean(convergence_metrics[, 4], na.rm = T), 2), nsmall = 2)

#Convergence Linear Beta Low 95%
results[1, 11] <- format(round(quantile(convergence_metrics[, 4], probs = 0.025, na.rm = T), 2), nsmall =
2)

#Convergence Linear Beta High 95%
results[1, 12] <- format(round(quantile(convergence_metrics[, 4], probs = 0.975, na.rm = T), 2), nsmall =
2)

#Making into A data frame
results <- as.data.frame(results)

#Column Names
colnames(results) <- c("Within_SD", "Within_SD_Low_95CI", "Within_SD_High_95CI",
            "Between_SD", "Between_SD_Low_95CI", "Between_SD_High_95CI",
            "Combined_SD", "Combined_SD_Low_95CI", "Combined_SD_High_95CI",
            "Linear_Beta", "Linear_Beta_Low_95CI", "Linear_Beta_SD_High_95CI")
```

```
  View(results)

}

bootMixedfunction_within_device_variance <-function(datainput){

  library(nlme)
  #Variance of Within and Between and Overall Model
  corr_model <- tryCatch(lme(bootdiff~ 1, random=~1|Athlete,
                    correlation=corCompSymm(form=~1|Athlete), data=datainput, na.action=na.omit),
                  error=function(e) e
  )

  # if error, go to next iteration
  if(inherits(corr_model, "error")) next

  bootwithinsd<-as.numeric(VarCorr(corr_model)[2,2])

  #Convergence Linear Mixed Model

  mixed_model <- lme(mean_Difference_Uplift ~ mean_Difference_Qualisys, random=~1|Athlete,
               data=datainput, na.action=na.omit)

  bootassociation <- as.numeric(summary(mixed_model)$tTable[2,1])

  combined <-cbind(bootwithinsd, bootassociation)
```

```
  combined

}



bootstrap_□unction_nm_within_device <- □unction(data□rame, N) {

  results <- matrix(nrow = 1,ncol = 6) #1 Row, columns □or bootstrap convergence

  convergence_metrics <- data.□rame() #Data □rame □or each bootstrap per□ormance metrics

  #Bootstrap □or loop
  □or(boot in 1:N){

    #Bootstrap Sample
    boot_sample <- data□rame[sample(nrow(data□rame), replace = T), ]



    #Variance o□Different Paramaters Between Difference o□COnstruct and Testing Technolgoy □or Elbow
Varus Torque

    bootwithinsd <- bootMixed□unction_within_device_variance(boot_sample)[1] #Within Pitcher SD

    bootassociation <- bootMixed□unction_within_device_variance(boot_sample)[2] #Association Just So I
can See it
```

```
  # if error, go to next iteration
  if(inherits(bootwithinsd, "error")) next

  if(inherits(bootassociation, "error")) next

  #Association



  convergence =  cbind(bootwithinsd, bootassociation)


  convergence = data.frame(convergence)


  convergence_metrics = rbind(convergence_metrics, convergence)


  print(paste("Progress:", boot, sep=""))


 }


 #Getting Aggregated Device Variance
 #Within SD Mean
 results[1, 1] <- format(round(mean(convergence_metrics[, 1], na.rm = T), 1), nsmall = 1)


 #Within SD Low 95%
 results[1, 2] <- format(round(quantile(convergence_metrics[, 1], probs = 0.025, na.rm = T) ,1), nsmall =
1)
```

```
#Within SD High 95%

results[1, 3] <- format(round(quantile(convergence_metrics[, 1], probs = 0.975, na.rm = T), 1), nsmall =
1)


#Association Linear Beta S

results[1, 4] <- format(round(mean(convergence_metrics[, 2], na.rm = T), 1), nsmall = 1)


#Association Linear Low 95%

results[1, 5] <- format(round(quantile(convergence_metrics[, 2], probs = 0.025, na.rm = T), 1), nsmall =
1)


#Association Linear High 95%

results[1, 6] <- format(round(quantile(convergence_metrics[, 2], probs = 0.975, na.rm = T), 1), nsmall =
1)



#Making into A data frame

results <- as.data.frame(results)


#Column Names

colnames(results) <- c("Within_SD", "Within_SD_Low_95CI", "Within_SD_High_95CI",

                 "Linear_Beta", "Linear_Beta_Low_95CI", "Linear_Beta_SD_High_95CI")


View(results)


}
```

```
###Data

Difference <- ROM_validity




#Limits of Agreement - Bland Altman


#Raw Nm

loa <- agree_nest(x = "Difference_Qualisys",
                  y = "Difference_Uplift",
                  id = "Athlete",
                  data = Difference,
                  agree.level = .80)

check(loa) #Checking Assumptions

#All are within acceptable limits


png("C:/Users/jduane/Documents/NEW WAVE/RSFMAE_BA.png", height = 1000, width = 2000, res = 300)

plot(loa, delta = 20)  #Maximum LOA of 20 nm (10nm each side).

dev.off()
```

```
#############################
#Convergence and Variance

#Function To Bootstrap Within and Total SD for Mixed Effects Models, For Validity of Convergence

##############################
#2000 Bootstraps for Within and Total Variance for Convergence Validity

#Set Seed
set.seed(17)

bootstrap_validity_function_nm(Difference, 2000)

######################
#Convergence Validity
model <- lmer(Difference_Uplift ~ Difference_Qualisys + (1|Athlete), data = Difference)
summary(model)
confint(model)

ggprediction <- predict_response(model, terms = c("Difference_Qualisys"))
```

```
png("file_path.png", height = 1000, width = 2000, res = 300)
plot(ggprediction, show_data = TRUE, jitter = T) +
  xlab("Qualisys RE") +
  ylab("Uplift RE") +
  labs(title = NULL)
dev.off()





####################
#Test Re-Test Reliability
data <- read_excel(file_path)

# Extract the columns for the two measurements
measurements <- data[, c("CMJ_Jump_1", "CMJ_Jump_2")]

# Calculate ICC
icc_result <- icc(measurements, model = "twoway", type = "agreement", unit = "average")
print(icc_result)


#Standard Error of Measure
calculate_sem(0.2, 0.93)
```

```
#Minimum Detectable Change

calculate_mdc(0.053)
```
